# Simulators with Haptic Feedback in Neurosurgery: Are We Reaching the “Aviator” Type of Training? Narrative Review and Future Perspectives

**DOI:** 10.3390/life15050777

**Published:** 2025-05-13

**Authors:** Davide Luglietto, Alessandro De Benedictis, Alessandra Marasi, Maria Camilla Rossi-Espagnet, Antonio Napolitano, Sergio Capelli, Vittorio Ricciuti, Daniele Riccio, Carlo Efisio Marras

**Affiliations:** 1 Neurosurgery Unit, Bambino Gesù Children’s Hospital, IRCCS, 00146 Rome, Italy; davide.luglietto@opbg.net (D.L.); alessandra.marasi@opbg.net (A.M.); carloefisio.marras@opbg.net (C.E.M.); 2 Department of Electrical Engineering and Information Technology (DIETI), University “Federico II”, 80131 Naples, Italy; daniele.riccio@unina.it; 3 Department of Electronic, Information, and Bioengineering, Politecnico di Milano, 20156 Milan, Italy; 4 Neuroradiology Unit, Bambino Gesù Children’s Hospital, IRCCS, 00146 Rome, Italy; mcamilla.rossi@opbg.net; 5 Medical Physics, Bambino Gesù Children’s Hospital, IRCCS, 00146 Rome, Italy; antonio.napolitano@opbg.net; 6 Division of Neurosurgery, Department of Medical and Surgical Specialties, Radiological Sciences and Public Health, University of Brescia, 25123 Brescia, Italy; s.capelli007@unibs.it; 7 Division of Neurosurgery, Department of Biotechnology and Life Sciences, University of Insubria, 21100 Varese, Italy; 8 School of Medicine and Surgery, University of Milano-Bicocca, 20900 Monza, Italy; v.ricciuti@campus.unimib.it; 9 Neurosurgery, Fondazione IRCCS San Gerardo dei Tintori, 20900 Monza, Italy

**Keywords:** haptics, neurosurgery, simulation, virtual reality, augmented reality, virtual reality

## Abstract

Over the last decade, the quality of neurosurgical procedures dramatically improved, also thanks to the development and increased accessibility of several technological recourses (e.g., imaging, neuronavigation, neurophysiology, microscopy), allowing to plan increasingly complex approaches, while reducing the risk of postoperative complications. Among these resources, three-dimensional rendering and simulation systems, such as virtual and augmented reality, provide a high-quality visual reconstruction of brain structures and interaction with advanced anatomical models. Although the usefulness of these systems is now widely recognized, the additional availability of proprioceptive (haptic) feedback might help to further enhance the realism of surgical simulation. A systematic literature review on the application of haptic technology in simulation of cranial neurosurgical procedures was made. Inclusion criteria were the usage of simulators with haptic feedback for specific neurosurgical procedures whereas the studies that did not include an evaluation of the surgical simulation system by a surgeon were excluded. According to inclusion and exclusion criteria, 10 studies were selected. Simulation in neurosurgery still lacks a system capable of rehearsing the entire procedure—from skin incision to skin closure—while providing both visual and proprioceptive feedback. Consequently, further advancements in this area are necessary.

## 1. Introduction

Neurosurgeons are faced with the imperative to develop both theoretical knowledge and practical skills from the onset of their residency. This knowledge is essential to face the complexities of neurosurgery, where the margin for error continues to diminish. In recent decades, advancements in technology have significantly enhanced the quality of neurosurgical practice, particularly through the implementation of simulation and three-dimensional rendering systems [1,2,3,4,5,6,7,8,9].

The advent of virtual reality (VR) and augmented reality (AR) has empowered both inexperienced and seasoned practitioners to explore scenarios where anatomical structures are either preserved or altered.

For novice students, these systems facilitate the study of anatomy, while for experienced professionals, they offer the ability to tailor surgical plans to individual cases, foresee potential challenges, and ensure the safest approach based on the patient’s unique anatomy.

In this context, few research groups have developed integrated systems combining visual reconstruction with haptic feedback, primarily for educational and pre-surgical purposes [10,11,12,13,14,15,16], in addition to discovering its potential in robotic neurosurgery [17].

As neurosurgical training evolves and the quest for optimal surgical outcomes while minimizing postoperative complications persists, the importance of simulation systems with haptic devices, similar to those employed in the aeronautical industry, continues to increase within neurosurgery.

Here, we conduct a narrative review, focusing on areas for the development and implementation of such systems in neurosurgical everyday practice.

## 2. Materials and Methods

**Figure 1:**
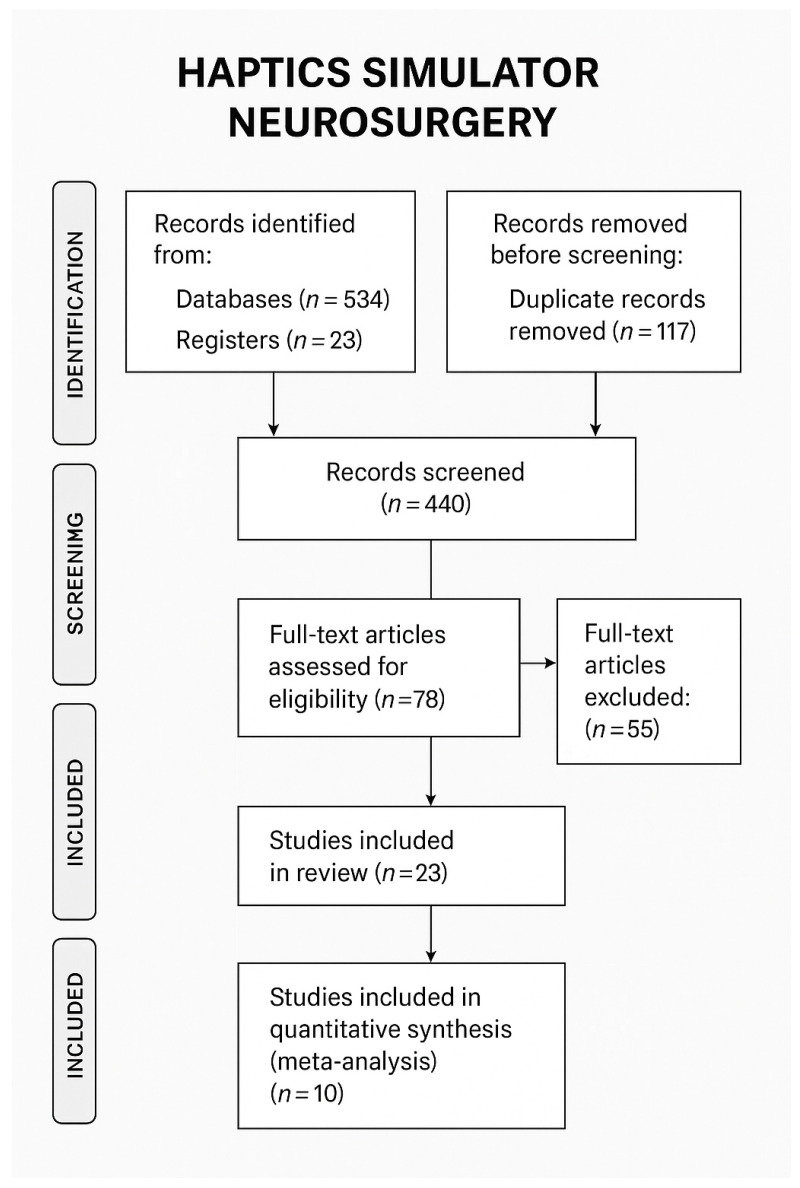
PRISMA flow diagram.

This systematic review followed the Preferred Reporting Items for Systematic Reviews and Meta-Analyses (PRISMA) 2020 guidelines. A comprehensive literature search was conducted across major databases, including PubMed, Scopus, and Web of Science, to identify studies related to the use of haptics and simulators in neurosurgery. A total of 534 records were identified through database searching, and an additional 23 records were obtained from other sources such as gray literature and reference list screening, resulting in 557 records. After removing 117 duplicates, 440 unique records remained for title and abstract screening. Of these, 362 records were excluded due to irrelevance to the study topic. The full texts of 78 articles were assessed for eligibility based on predefined inclusion and exclusion criteria. The inclusion criteria were the usage of simulators with haptic feedback for specific neurosurgical procedures. Exclusion criteria were narrative reviews and studies that did not include an evaluation of the surgical simulation system by a surgeon (or medical student or resident). A total of 55 full-text articles were excluded, primarily for not meeting the criteria related to simulation use, neurosurgical focus, or relevance to haptic technology. Ultimately, 23 studies met the inclusion criteria and were included in the qualitative synthesis. Of these, 10 studies provided sufficient quantitative data and were included in the meta-analysis (Figure 1).

## 3. Results

The simulators described in the literature display a high degree of diversity and significant potential for neurosurgical training (Table 1). While current systems still fall short in replicating the complete microsurgical workflow—particularly due to missing anatomical details and omitted surgical steps—they nonetheless enable neurosurgeons to visualize patient-specific 3D brain models and engage with realistic interaction forces between virtual brain tissue and surgical instruments. Most simulation platforms consist of a visual interface, a bimanual haptic feedback system, and a high-performance computing unit. Each study typically focuses on the execution and refinement of a specific surgical maneuver. For instance, a virtual aneurysm-clipping simulator has been developed, featuring haptic force feedback, real-time vessel deformation, and simulated blood flow, aimed at improving technical precision.

Simulation systems have been evaluated by both neurosurgical trainees and experienced surgeons. Evidence suggests that haptic simulation training significantly enhances operative performance in endoscopic neurosurgery [14]. Additionally, integrated systems like NeuroTouch (National Research Council, Ottawa, ON, Canada) enable the quantification of tool-tissue interaction forces and psychomotor metrics, facilitating performance assessment. These virtual platforms, particularly those incorporating haptic technology, have shown utility in improving tactile discrimination—crucial for microsurgical technique—and are increasingly used to evaluate motor and sensory competencies in neurosurgery residency candidates.

Simulators have been implemented in multiple clinical training centers, with advancements including realistic graphical rendering, tissue deformation models, and improved haptic feedback. However, a common limitation across studies remains the restricted scope of simulation, often confined to isolated surgical steps rather than representing the full surgical procedure.

## 4. Discussion

### 4.1. Principles of Simulation in Neurosurgery

Research in surgical simulation has evolved over recent years, leading to the development of various systems with diverse characteristics. Malone et al. have provided a detailed description of all neurosurgically relevant simulation systems [3].

These systems can be categorized into two main groups: those that train specific parts of a procedure, such as ventricular puncture [22,23], and those that simulate entire procedures, recreating an environment comparable to a real operating room (e.g., NeuroTouch system) [11].

In neurosurgery, the availability of preoperative volumetric imaging enhances simulation resources, providing clearer representations of anatomical relationships and pathological alterations.

Anatomical structures and their spatial interactions can be reconstructed from preoperative imaging using image segmentation algorithms [24].

Segmentation can be used for different neurosurgical pathologies and consequent practical implications. Several studies described its application in the field of epileptology, such as the detection of focal cortical dysplasias using neural network systems [25].

**Figure 2:**
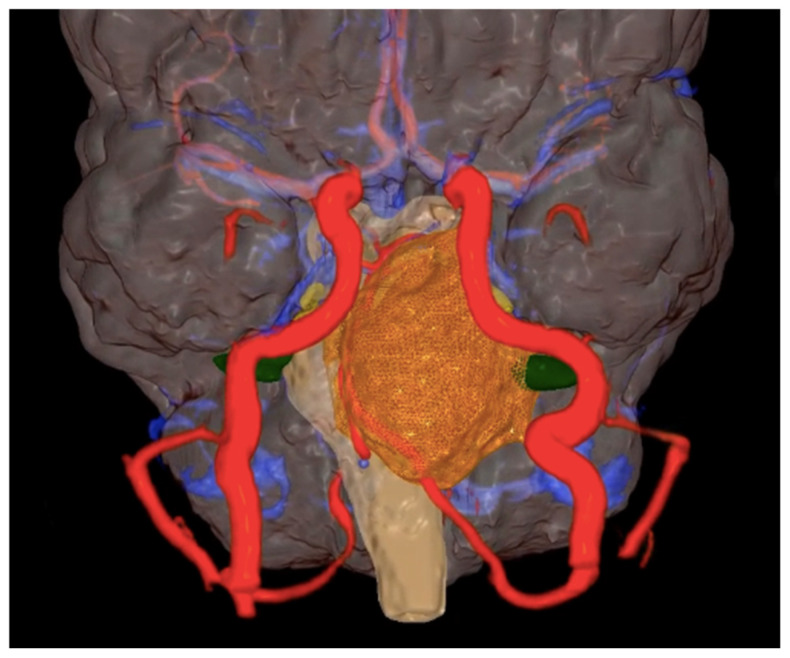
Notably, 3D Preoperative model in a posterior fossa lesion.

The concept of image segmentation is also applied to the reconstruction of space-occupying lesions, where defining the pathological area allows for identifying the boundaries of resection and associated risks (Figure 2) [26].

Moreover, for intracranial aneurysms, the MATCH study research groups defined the most popular segmentation algorithms for intracranial aneurysms in 3D DSAs datasets [27].

But, when we talk about automatic segmentation there are several limitations: in particular, errors from automatic segmentation often necessitate manual correction or semi-automatic systems where technicians outline anatomical margins while algorithms refine models’ morphology [28,29,30].

To enhance realism, it is necessary to consider additional physical elements, including biomechanical properties and optical factors. As highlighted by several experimental studies, the mechanical response of the brain to touch is a complex mechanism with a highly nonlinear stress-strain relationship, and tissue stiffness in compression is much higher than in extension [31,32].

The complex mechanical behavior of the brain necessitates careful selection of constitutive models for specific applications. The choice of constitutive model in surgical simulations depends on the characteristic strain rate of the process and computational efficiency considerations. Fortunately, intraoperative image registration, akin to a Dirichlet-type problem, does not require precise knowledge of patient-specific mechanical properties, as demonstrated in previous studies [33].

Therefore, biomechanical properties may be empirically determined from models but prove limited in generating pathological models with varied characteristics [6,34].

Optical properties, including light absorption and surface reflectance, pose complex computational challenges, especially when aiming for visually realistic rendering resembling a surgeon’s field of view [35,36].

Once developed, 3D models are versatile and can be integrated into VR (virtual reality) and AR (augmented reality) systems facilitating user interaction by the operator.

VR, AR, and MR are distinct technologies. VR entails the creation of immersive three-dimensional (3D) computer-generated environments, while AR involves overlaying computer-generated images onto real-world visuals. On the other hand, MR projects virtual objects into the real world, with these objects possessing spatial awareness and responsiveness [37,38].

**Figure 3:**
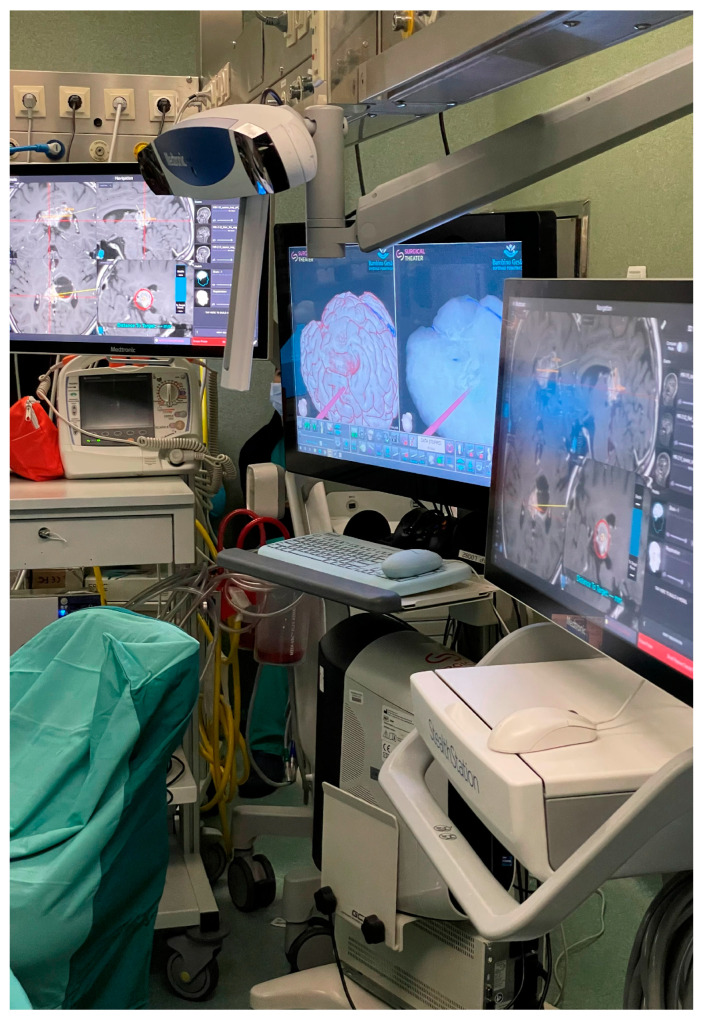
Surgical Theater VR Station, operative setting.

In neurosurgery, VR tools are available both for educational purposes and surgical planning (Figure 3) [38,39].

Some examples of surgical planning VR devices are Surgical Theater (Beachwood, OH, USA), Dextroscope (Volume Interactions Pte Ltd., Singapore), VPI Reveal (Eindhoven, North Brabant, The Netherlands), and Synaptive Medical (Synaptive Medical Inc., Toronto, OT, Canada).

On the other hand, augmented reality (AR) systems, such as Microsoft’s HoloLens2 (Microsoft Corporation, Redmond, WA, USA), facilitate user interaction with 3D models via hand gestures and holographic buttons [40]. This is a Head-Mounted Device (HMD), that mounts the Windows 10 operating system and represents an improvement over the first type of HoloLens.

The user can associate a set of scripts with each 3D model, thus allowing user interaction (grab, rotate, enlarge, and shrink) with objects, controlled by hand gestures and their relative inputs via holographic buttons.

**Figure 4:**
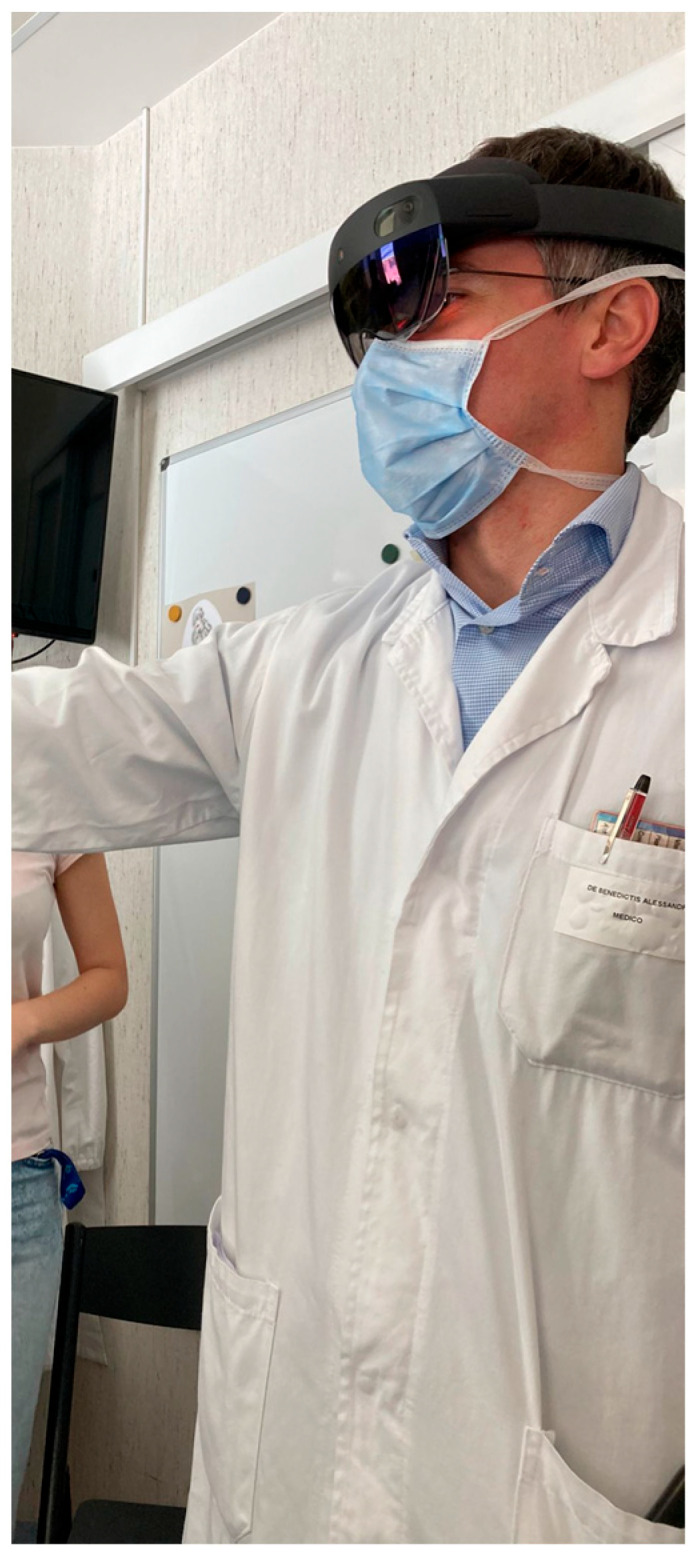
Microsoft HoloLens2 testing for anatomical models.

Moreover, 3D structures can be displayed or hidden by specific functions based on changing a Boolean variable when the user presses the corresponding button (Figure 4) [7].

Haptic interfaces, on the other hand, create a tactile connection between operators and simulation objects. Haptic interaction is pivotal in VR systems for its capacity to facilitate realistic interactions between the operator and virtual objects [41]. This aspect significantly enhances the immersion of virtual experiences and finds valuable applications in virtual training. From a technical perspective, haptic interaction primarily encompasses tactile sensing and haptic feedback. Tactile sensing devices are adept at capturing touch characteristics through proximity or contact between the human hand and objects. Tactile sensors serve two primary functions: sensing and recognition. They can detect various contact conditions and even multidimensional forces [41,42]. Additionally, tactile sensors can gather information such as the physical characteristics of objects (e.g., smoothness, hardness, texture, and shape) and operational statuses (e.g., contact, friction, and slippage) [7,8,9]. This functionality enables real-time monitoring of the interaction process between robot hands and objects, facilitating efficient virtual control or robot teleoperation [10,11,12].

Haptic systems require between 3 and 6 degrees of movement to accurately reproduce translational and rotational movements of instruments, with grasping and cutting motions requiring additional degrees of movement. As systems become more complex, user perception of transparency is reduced. Some systems also incorporate piezoelectric transducers to reproduce vibration sensations, as seen in the use of microsurgical drills [16,43]. Techniques ensuring stable tactile interaction with complex geometries have been introduced recently, but methods for realistic manipulation of deformable objects are still underdeveloped. The challenge for tactile rendering lies in the accurate coupling of simulation with haptic interfaces in a closed loop with the human operator, ensuring stability and responsiveness. The fidelity or even lack of tactile feedback is one of the main limitations of current simulation systems, but perhaps recent advancements can be integrated into neurosurgical simulators to address this issue [11,13,14].

In terms of hardware for graphic display, the gaming industry has facilitated the spread of visually haptic display systems that are easy to use and cost-effective, potentially allowing easy access to simulation systems for trainees and new specialists in the future.

### 4.2. Neurosurgical Simulators with Haptic Feedback: State of the Art

In neurosurgery, there are few examples in the literature of simulation systems with haptic feedback [11,13,14,15,16,20]. These mainly fall into the group of step-wise simulators built to improve specific skills. Some of these systems, such as NeuroTouch, have been progressively implemented for specialist training [11].

Subsequent studies have validated specific execution capabilities of certain tasks using these simulation systems, even suggesting their use in neurosurgical residency selection processes [18,19,44]. The main components of these neurosurgical simulators include a 3-dimensional graphics rendering system (stereoscope), a bimanual haptic rendering system, additional controls, and 1 or 2 computers. These simulators are mounted on a frame that allows adjustment of height and tilt angles. The graphics rendering system mimics a neurosurgical microscope, while the haptic devices include the Phantom Desktop and Freedom 6S, each offering different degrees of freedom for tactile interaction.

NeuroTouch, a virtual simulator equipped with haptic feedback, is specifically designed to facilitate the acquisition and evaluation of technical skills essential for craniotomy-based procedures. Prototypes have been established in seven teaching hospitals across Canada for beta testing and validation, as well as to assess the integration of NeuroTouch-simulated surgery into neurosurgery training curricula [14,15].

The simulator is built to examine two different tasks: tumor debulking and tumor cauterization. In the tumor debulking task, the simulation can be performed only with one hand, holding either the aspirator or the ultrasonic aspirator or with both hands each holding one tool.

The tumor cauterization task is meant to be performed with the bipolar electrocautery in the dominant hand and the aspirator in the nondominant hand.

After the collection of comments by users and tutors, the most acclaimed advantage of the NeuroTouch system was the visual feedback whereas the most criticized one was touch.

Therefore, the system still needs some improvements: implementation of finer grasping of tissues, blunt and sharp dissection, extending the set of tools, the ability to rapidly change these tools during a training scenario and development of further tasks. Eventually, the NeuroTouch could potentially be extended to patient-specific rehearsal through the conversion of medical images of patients into simulation models.

Other examples of simulators with haptic systems have been individually adapted to specific needs, such as a simulation system for aneurysm clipping [13]. In this case, a 3D model of aneurysm geometry was reconstructed from angioCT, incorporating vessel deformability and haptic feedback force calculations. Additionally, a blood flow simulation system was implemented, modeling blood as an incompressible Newtonian fluid using computational methods on parallel GPU architectures [45].

This study marks the inaugural utilization of original clipping forceps in virtual aneurysm-clipping simulations, accompanied by real-time haptic feedback. This adaptation markedly enhances the realism of the virtual aneurysm-clipping experience. As the clipping forceps gradually close, the coordinated use of a second hand holding the suction tip may refine or stabilize the clip’s positioning through gentle manipulation of the aneurysm. Additionally, the simulation realistically replicates the necessity for depth perception and hand-eye coordination when maneuvering the forceps towards the aneurysm.

A third of the users of this simulator described the haptic interaction with the vessels as adequate, although the simulation and the clipping procedure itself still need technical improvement, as rated not realistic by a minority of participants.

Another example of the haptic simulator is described in the study by Ghasemloonia et al. [16], where a haptic simulation system was designed for simulating temporal bone drilling.

This study involved data collection using vibration signals, post-processing with Matlab, and rendering 3D acceleration signals to provide real-time haptic feedback using different haptic devices. Due to differing force and bandwidth characteristics among haptic interfaces, four commercially available devices at various price points were assessed. Each device’s ability to render vibration feedback was evaluated by recording output acceleration signals at the end-effector. These recorded accelerations were quantitatively compared in both time and frequency domains through coherence and cross-correlation analyses. Based on their rendering capacities under different drilling conditions, the devices were ranked. Results showed that the Phantom Desktop (V3.1) and the Phantom Omni (v5.1.9) outperformed the Falcon and the Omega 7 (v4.0.28) in effectively rendering vibration signals in both time and frequency domains. In this case, future development is needed to add vibrotactile rendering to surgical platforms for surgeries that include drilling.

In conclusion, these methodologies remain isolated and have not yet been standardized for educational or preoperative use.

### 4.3. Future Perspectives

**Figure 5:**
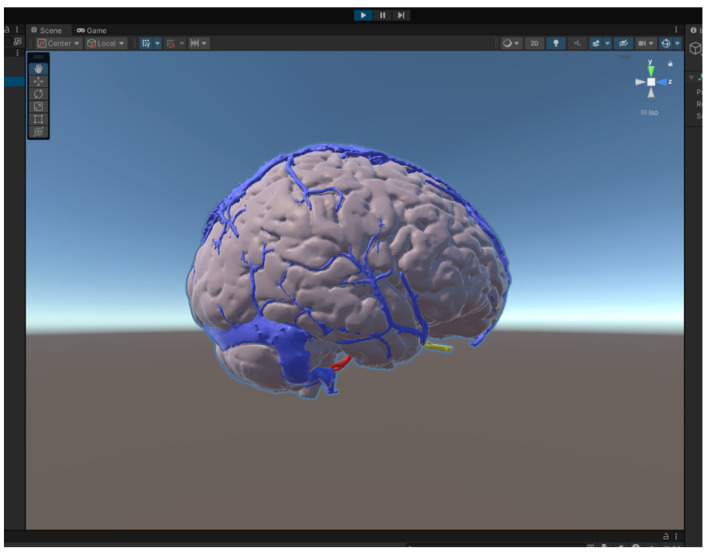
Brain model on Unity 3D software (v.6.1.1).

3D reconstructions exported into a VR system are routinely used in most centers for preoperative planning (Figure 5). DICOM sequences of anatomical structures can be processed using Mimics Medical v.25.0 software and different segmentation tools are applied to create regions of interest (ROIs), which are then converted into STL files for integration into a variable VR system.

In order to build an integrated system where AR can also benefit from haptic interaction, the future goal is firstly to conceptualize the perceived force during various phases of a neurosurgical cranial approach: skin incision, high-speed drill usage (craniotomy), dural opening, corticectomy, and variations in tactile perception between healthy and pathological tissue. Secondly, to reconstruct the frictional force for each sensory event and integrate these models with haptic devices for augmented reality visualization.

The final step will eventually be to build a virtual operating room in AR integrating cranial models and surgical instruments for educational purposes.

From the analysis of the literature, it emerged that the development of simulation systems with haptic feedback in neurosurgery can help, on the one hand, less experienced surgeons to gain experience in a safer asset while the more experienced ones predict any critical issues that may arise found in the operating room.

The greatest critical issues are currently found in the development of systems that can realistically reconstruct the hand-tool, tool-object interaction and the variations in the consistency of the object (both in the various phases of a procedure and inter-individual with respect to healthy or pathological tissue).

## 5. Conclusions

Three-dimensional rendering and simulation systems, such as virtual and augmented reality, offer high-quality visual reconstructions of brain structures and enable interaction with advanced anatomical models. While the utility of these systems is widely acknowledged, the incorporation of proprioceptive (haptic) feedback, as already demonstrated, could further enhance the realism of surgical simulation and standardize its usage for surgical rehearsal and educational purposes.

In conclusion, neurosurgical simulation still lacks a system capable of rehearsing the entire procedure—from skin incision to skin closure—while providing both visual and proprioceptive feedback. Consequently, further advancements in this area are necessary.

## Figures and Tables

**Table 1:** Selected articles with a specific focus on haptic simulation and neurosurgery.

First Name and Year of Publication	Article Type	Focus	Simulator System Components	Evaluation Criteria	Advantages	Limits	Results
Su (2020) [15]	Original Article	Ventricular puncture training	Virtual surgical scene and haptic device of Geomagic Touch X (3D Systems, Rock Hill, CA, USA), 3D printed brain model	Questionnaire for 3 different groups: training ventricular puncture on a 3D printed model after virtual rehearsal with haptic feedback (1st group), without haptic feedback (2nd group), without any type of simulation	Both virtual simulators with haptic feedback and 3D-printed models	Single step of a procedure	Neurosurgeons can visualize a 3D virtual human brain and experience realistic interaction forces between the virtual brain tissue and virtual instruments. Experimental results have shown that the proposed simulator effectively enhances neurosurgeons’ lateral ventricle puncture operation skills.
Gmeiner (2018) [13]	Original Article	Aneurysm surgery training	Stereovision system, bimanual haptic tool manipulators, high-end computer	Evaluated by 18 neurosurgeons. In 4 patients with different medial cerebral artery aneurysms, virtual clipping was performed after real-life surgery, and surgical results were compared regarding clip application, surgical trajectory, and blood flow.	Bimanual simulation with 2 instruments, blood flow simulation, clip positioning simulation	Missing simulation of arachnoid dissection and vascular manipulation around the aneurysm	A patient-specific virtual aneurysm-clipping simulator featuring haptic force feedback and real-time deformation of vessels and aneurysms has been developed. This simulation software also includes an integrated evaluation of the clipping procedure through blood flow simulation.
Micko (2017) [18]	Original Article	Impact of sleep interruption on neurosurgical performance	Stereovision system, bimanual haptic tool manipulators, high-end computer	Medical students and residents evaluated performing an identical microsurgical task, well rested (baseline test), and after sleep interruption at night (stress test)	Evaluating the effects of fatigue on surgical performance	Participants were not randomized during baseline and stress tests because of planning of on-calls	Increase of neurosurgical simulator performance in neurosurgical residents and medical students under simulated night shift conditions.
Thawani (2016) [14]	Clinical Study	Effect of simulation training on performance in the operating room	Stereovision system, bimanual haptic tool manipulators, high-end computer	Subjects were divided into two groups, an experimental group that underwent instructed simulation training sessions, and a control group that did not undergo training. Subjects were evaluated by an expert based on their performance in two simulated sessions (before and after training), and intra-operatively using a VAS scale with six independent measures. The evaluator was blinded to the trained/ untrained assignment for each subject.	Evaluation of intraoperative performance after simulation.	Small number of subjects and adjudication bias	The data suggest that haptic simulation training in endoscopic neurosurgery can enhance operative performance.
Ghasemloonia (2016) [16]	Original Article	Temporal bone drilling simulation	Cadaveric temporal bone dissection rendered through a haptic interface, different haptic devices rendering vibrotactile feedback.	The same accelerometer that was used to record hevibration of the surgical drill was mounted on each of these devices to record the output vibration signal.	Evaluation after acquired data with cadaveric drilling	Single step of a procedure	Vibration data from cadaveric temporal bone dissections were collected by recording the accelerations of the surgical drill, and the signals were post-processed for rendering on a variety of haptic displays.
Alotaibi (2015) [19]	Research Article	NeuroTouch training system	Stereoscopic viewer, bimanual force feedback handles, and activator pedal. Mannequin head with suction instrument.	Metrics direct from CSV files of NeuroTouch data output	Standardized metrics be useful in developing multi-institutional databases from centers utilizing the NeuroTouch platform	Not applicable	Data extraction from NeuroTouch system to track and compare psychomotor performance.
Patel (2014) [20]	Research Article	Immersive touch simulation system	Immersive touch virtual simulator, haptic articulating arm, bipolar and suction tools used to feel the virtual spheres.	Group A did a simulation exercise prior to the task involving the brain cavity model, subjects in Group B were excluded from the virtual simulation exercise.	Complete surgical task with virtual rehearsal followed by model simulation	Not applicable	Virtual computer-based simulators with integrated haptic technology may improve tactile discrimination required for microsurgical technique.
Roitberg (2013) [21]	Pilot study	Haptic neurosurgical simulator	Three-dimensional surgical simulator with head and arm tracking, collocalization, and haptic feedback.	Neurosurgical residents in two cohorts (year 1 and year 2)	Measurements of sensory-motor skills in an objective and reproducible way, set of 3 tests of sensory-motor function.	Not applicable	Use of a virtual reality simulator as a tool to measure motor and sensory performance in applicants to a neurosurgery residency. The results support the hypothesis that the combined performance score measures a trait that has a bell-curve distribution in the population.
Delorme (2012) [11]	Operative Technique	NeuroTouch simulator for cranial microneurosurgery	Stereovision system, bimanual haptic tool manipulators, high-end computer	Questionnaire of an Advisory Network of Teaching Hospitals in Canada (6-year neurosurgery resident)	Realistic 3d graphics combined with the haptic feedback and the stereo vision system.	Focuses only on tumor debulking and tumor cauterization	Two training tasks were implemented for practicing skills with 3 different surgical tools. This system was implemented across 7 hospitals in Canada.
Lemole (2007) [22]	Research Article	Immersive touch simulation system	Immersive touch virtual simulator, haptic articulating arm, bipolar and suction tools used to feel the virtual spheres.	Not evaluated	First example of surgical simulator with haptic feedback	Focuses only on ventriculostomy placement module, inability to detect and register force feedback for sidewall collisions.	The simulation platform was found to have realistic visual, tactile, and handling characteristics, as assessed by neurosurgical faculty, residents, and medical students.

## Data Availability

No new data were created or analyzed in this study. Data sharing is not applicable to this article.

## References

[1] Rehder R., Abd-El-Barr M., Hooten K., Weinstock P., Madsen J.R., Cohen A.R. (2015). The role of simulation in neurosurgery. Child’s Nerv. Syst..

[2] Thawani J., Randazzo M., Pisapia J., Singh N. (2016). 3D printing in neurosurgery: A systematic review. Surg. Neurol. Int..

[3] Malone H.R., Syed O.N., Downes M.S., D’Ambrosio A.L., Quest D.O., Kaiser M.G. (2010). Simulation in neurosurgery: A review of computer-based simulation environments and their surgical applications. Neurosurgery.

[4] Spacca B., Luglietto D., Vatavu O., D’Incerti L., Tuccinardi G., Butti D., Bussolin L., Mussa F., Genitori L. (2023). Operational Improvement in Pediatric Neurosurgery. Frailty in Children: From the Perioperative Management to the Multidisciplinary Approach.

[5] You Y., Niu Y., Sun F., Huang S., Ding P., Wang X., Zhang X., Zhang J. (2022). Three-dimensional printing and 3D slicer powerful tools in understanding and treating neurosurgical diseases. Front. Surg..

[6] Miller K., Joldes G.R., Bourantas G., Warfield S.K., Hyde D.E., Kikinis R., Wittek A. (2019). Biomechanical modeling and computer simulation of the brain during neurosurgery. Int. J. Numer. Methods Biomed. Eng..

[7] De Benedictis A., Marasi A., Rossi-Espagnet M.C., Napolitano A., Parrillo C., Fracassi D., Baldassari G., Borro L., Bua A., de Palma L. (2023). Vertical Hemispherotomy: Contribution of Advanced Three-Dimensional Modeling for Presurgical Planning and Training. J. Clin. Med..

[8] Mussi E., Mussa F., Santarelli C., Scagnet M., Uccheddu F., Furferi R., Volpe Y., Genitori L. (2020). Current practice in preoperative virtual and physical simulation in neurosurgery. Bioengineering.

[9] Premuselli R., D’Amore C., Barba M., Marasi A., Del Baldo G., DE Benedictis A., Piccirilli E., Colafati G.S., Mastronuzzi A., Marras C.E. (2024). Operator perceived advantage of virtual surgical rehearsal in pediatric neurosurgical oncology: A preliminary experience. J. Neurosurg. Sci..

[10] L’orsa R., Macnab C.J., Tavakoli M. (2013). Introduction to Haptics for Neurosurgeons. Neurosurgery.

[11] Delorme S., Laroche D., DiRaddo R., Del Maestro R.F. (2012). NeuroTouch: A physics-based virtual simulator for cranial microneurosurgery training. Neurosurgery.

[12] Joseph F.J., Vanluchene H.E.R., Bervini D. (2023). Simulation training approaches in intracranial aneurysm surgery—A systematic review. Neurosurg. Rev..

[13] Gmeiner M., Dirnberger J., Fenz W., Gollwitzer M., Wurm G., Trenkler J., Gruber A. (2018). Virtual Cerebral Aneurysm Clipping with Real-Time Haptic Force Feedback in Neurosurgical Education. World Neurosurg..

[14] Thawani J.P., Ramayya A.G., Abdullah K.G., Hudgins E., Vaughan K., Piazza M., Madsen P.J., Buch V., Grady M.S. (2016). Resident simulation training in endoscopic endonasal surgery utilizing haptic feedback technology. J. Clin. Neurosci..

[15] Su X.H., Deng Z., He B.W., Liu Y.Q. (2020). Haptic-based virtual reality simulator for lateral ventricle puncture operation. Int. J. Med. Robot. Comput. Assist. Surg..

[16] Ghasemloonia A., Baxandall S., Zareinia K., Lui J.T., Dort J.C., Sutherland G.R., Chan S. (2016). Evaluation of haptic interfaces for simulation of drill vibration in virtual temporal bone surgery. Comput. Biol. Med..

[17] Aggravi M., De Momi E., DiMeco F., Cardinale F., Casaceli G., Riva M., Ferrigno G., Prattichizzo D. (2015). Hand–tool–tissue interaction forces in neurosurgery for haptic rendering. Med. Biol. Eng. Comput..

[18] Micko A., Knopp K., Knosp E., Wolfsberger S. (2017). Microsurgical Performance After Sleep Interruption: A NeuroTouch Simulator Study. World Neurosurg..

[19] Alotaibi F.E., AlZhrani G.A., Sabbagh A.J., Azarnoush H., Winkler-Schwartz A., Del Maestro R.F. (2015). Neurosurgical Assessment of Metrics Including Judgment and Dexterity Using the Virtual Reality Simulator NeuroTouch (NAJD Metrics). Surg. Innov..

[20] Patel A., Koshy N., Ortega-Barnett J., Chan H.C., Kuo Y.-F., Luciano C., Rizzi S., Matulyauskas M., Kania P., Banerjee P. (2014). Neurosurgical tactile discrimination training with haptic-based virtual reality simulation. Neurol. Res..

[21] Roitberg B.Z., Kania P., Luciano C., Dharmavaram N., Banerjee P. (2015). Evaluation of Sensory and Motor Skills in Neurosurgery Applicants Using a Virtual Reality Neurosurgical Simulator: The Sensory-Motor Quotient. J. Surg. Educ..

[22] Lemole G.M., Banerjee P.P., Luciano C., Neckrysh S., Charbel F.T. (2007). Virtual reality in neurosurgical education: Part-task ventriculostomy simulation with dynamic visual and haptic feedback. Neurosurgery.

[23] Bradley P. (2006). The history of simulation in medical education and possible future directions. Med. Educ..

[24] Niessen W. (2008). Model-based image segmentation for image-guided interventions. Image-Guided Interventions: Technology and Applications.

[25] House P.M., Kopelyan M., Braniewska N., Silski B., Chudzinska A., Holst B., Sauvigny T., Martens T., Stodieck S., Pelzl S. (2021). Automated detection and segmentation of focal cortical dysplasias (FCDs) with artificial intelligence: Presentation of a novel convolutional neural network and its prospective clinical validation. Epilepsy Res..

[26] Upreti G. (2023). Advancements in Skull Base Surgery: Navigating Complex Challenges with Artificial Intelligence. Indian J. Otolaryngol. Head Neck Surg..

[27] Berg P., Voß S., Saalfeld S., Janiga G., Bergersen A.W., Valen-Sendstad K., Bruening J., Goubergrits L., Spuler A., Cancelliere N.M. (2018). Multiple Aneurysms AnaTomy CHallenge 2018 (MATCH): Phase I: Segmentation. Cardiovasc. Eng. Technol..

[28] Riener R., Harders M. (2012). Medical Model Generation. Virtual Reality in Medicine.

[29] Falcão A.X., Udupa J.K. (2000). A 3D generalization of user-steered live-wire segmentation. Med. Image Anal..

[30] Heckel F., Konrad O., Hahn H.K., Peitgen H.-O. (2011). Interactive 3D medical image segmentation with energy-minimizing implicit functions. Comput. Graph..

[31] Miller K., Chinzei K. (2002). Mechanical properties of brain tissue in tension. J. Biomech..

[32] Zhang C., Liu C., Zhao H. (2022). Mechanical properties of brain tissue based on microstructure. J. Mech. Behav. Biomed. Mater..

[33] Wittek A., Hawkins T., Miller K. (2008). On the unimportance of constitutive models in computing brain deformation for image-guided surgery. Biomech. Model. Mechanobiol..

[34] Miller K. (1999). Constitutive model of brain tissue suitable for finite element analysis of surgical procedures. J. Biomech..

[35] Preim B., Botha C.P. (2014). Visual Computing for Medicine: Theory, Algorithms, and Applications.

[36] Ropinski T., Doring C., Rezk-Salama C. Interactive volumetric lighting simulating scattering and shadowing. Proceedings of the 2010 IEEE Pacific Visualization Symposium (PacificVis 2010).

[37] Chan S., Conti F., Salisbury K., Blevins N.H. (2013). Virtual Reality Simulation in Neurosurgery. Neurosurgery.

[38] Mishra R., Narayanan K., Umana G.E., Montemurro N., Chaurasia B., Deora H. (2022). Virtual Reality in Neurosurgery: Beyond Neurosurgical Planning. Int. J. Environ. Res. Public Health.

[39] Lee C., Wong G.K.C. (2019). Virtual reality and augmented reality in the management of intracranial tumors: A review. J. Clin. Neurosci..

[40] Palumbo A. (2022). Microsoft HoloLens 2 in Medical and Healthcare Context: State of the Art and Future Prospects. Sensors.

[41] Kim J.J., Wang Y., Wang H., Lee S., Yokota T., Someya T. (2021). Skin Electronics: Next-Generation Device Platform for Virtual and Augmented Reality. Adv. Funct. Mater..

[42] Fang H., Guo J., Wu H. (2022). Wearable triboelectric devices for haptic perception and VR/AR applications. Nano Energy.

[43] McMahan W., Kuchenbecker K.J. Haptic display of realistic tool contact via dynamically compensated control of a dedicated actuator. Proceedings of the 2009 IEEE/RSJ International Conference on Intelligent Robots and Systems (IROS 2009).

[44] Gélinas-Phaneuf N., Choudhury N., Al-Habib A.R., Cabral A., Nadeau E., Mora V., Pazos V., Debergue P., DiRaddo R., Del Maestro R.F. (2013). Assessing performance in brain tumor resection using a novel virtual reality simulator. Int. J. Comput. Assist. Radiol. Surg..

[45] Fenz W., Dirnberger J., Georgiev I. Blood flow simulations with application to cerebral aneurysms. Proceedings of the 2016 Spring Simulation Multi-Conference.

